# Minimal Movement Restrictions Do Not Increase Hip Dislocations Following Total Hip Arthroplasty: A Before-and-After Study of 10,357 Patients

**DOI:** 10.2106/JBJS.25.00121

**Published:** 2026-02-19

**Authors:** Amanda D. Klaassen, Nienke W. Willigenburg, Jan Willem Q. Musters, Jelmer Jager, Rudolf W. Poolman, the Santeon Hip Osteoarthritis Group, Laurens Kaas

**Affiliations:** 1 Joint Research, Department of Orthopedic Surgery, OLVG Hospital, Amsterdam, The Netherlands; 2 Department of Orthopedics, Leiden University Medical Center, Leiden, The Netherlands; 3 Faculty of Health, University of Applied Sciences Leiden, Leiden, The Netherlands; 4 Department of Anesthesiology, Erasmus Medical Center, Rotterdam, The Netherlands.

## Abstract

**Background:**

Hip dislocation is the most common reason for early revision after total hip arthroplasty (THA). To prevent dislocation, patients are often prescribed movement restrictions following THA; however, these restrictions can be uncomfortable and limiting for patients and may delay a return to daily activities. The present study aimed to investigate whether a protocol with reduced movement restrictions following THA would lead to an increased incidence of early hip dislocations.

**Methods:**

In this uncontrolled, multicenter, before-and-after study, 2 groups were retrospectively compared. Patients who underwent a THA between January 1, 2015, and March 31, 2020, were included. Patients in the first group (n = 7,666) were prescribed strict movement restrictions following THA, and patients in the second group (n = 2,691) were prescribed minimal movement restrictions. Patient and prosthesis characteristics were collected, as well as data regarding hip dislocations within 90 days postoperatively as the primary outcome.

**Results:**

The incidence of early hip dislocations was not significantly different between movement groups, with 112 (1.46%) of 7,661 hips in the restricted group and 52 (1.93%) of 2,691 hips in the minimally restricted group experiencing a dislocation (p = 0.093).

**Conclusions:**

The use of a protocol with minimal movement restrictions following THA did not increase the incidence of early hip dislocations.

**Level of Evidence:**

Therapeutic Level III. See Instructions for Authors for a complete description of levels of evidence.

Hip dislocation is the most common reason for early revision following total hip arthroplasty (THA) and substantially affects patient physical function and emotional well-being^1,2^. Additionally, dislocations often require surgical revision and substantially impact hospital costs^3,4^. Reported dislocation rates vary following THA, with the average being around 2%^4-6^. Given that THA is one of the most frequently performed orthopaedic procedures worldwide, this percentage translates into a substantial number of complications. In 2022, around 36,700 primary THAs were performed in The Netherlands, with a total of 3,592 revisions (625 for a dislocation)^1^.

Soft-tissue damage from primary THA might make the hip joint more prone to dislocation during specific movements^7^. Traditionally, movement restrictions such as walking with crutches, sleeping supine, and using assistive devices to go to the toilet or put on socks are prescribed postoperatively, with the aim of reducing the risk of dislocation. However, these restrictions can be uncomfortable for patients and are associated with increased health-care costs and a delay in returning to normal activity^8-10^. Studies have shown that patients without such movement limitations tend to report higher satisfaction with the pace of their recovery and are able to independently perform activities of daily living sooner^8,11,12^. Evidence supporting the effectiveness of movement restrictions to prevent dislocations is limited. The literature suggests that instructions that remove or reduce hip precautions do not increase the number of dislocations^6,11-16^.

Despite growing evidence, orthopaedic surgeons and other clinicians remain reluctant to discontinue the prescription of movement restrictions following THA, regardless of the surgical approach^17-19^. Detecting significant differences in dislocation incidence between patients with strict and with minimal movement restrictions requires large sample sizes, particularly when accounting for variables such as surgical approach and head size.

Therefore, the aim of the present study was to investigate the incidence of early hip dislocations, within 90 days after THA, before and after the implementation of a protocol with minimal movement restrictions, in a large multicenter setting.

## Materials and Methods

### Patients and Design

In this uncontrolled, multicenter, before-and-after study, the incidence of early dislocation was compared between patients who received strict versus minimal movement restrictions following THA^20^. The study was approved by the Santeon institutional review board and by all local ethics committees of the participating hospitals (SDB-2021-002) and was conducted according to the Declaration of Helsinki. All data were completely anonymized before processing and analysis.

Patients were included who underwent primary THA for osteoarthritis (OA) between January 1, 2015, and March 31, 2020, in 1 of the 7 participating Santeon hospitals in The Netherlands (OLVG, Amsterdam; Canisius Wilhelmina Hospital, Nijmegen; Catharina Hospital, Eindhoven; Maasstad Hospital, Rotterdam; Martini Hospital, Groningen; Medisch Spectrum Twente, Enschede; and St. Antonius Hospital, Utrecht). Exclusion criteria were an age of <18 years and any diagnosis other than hip OA. In cases in which a patient underwent THA on both sides within 6 months, the first hip was included in the analysis and the second hip was excluded.

### Movement Restrictions

When patients participating in the value-based healthcare (VBHC) hip OA program expressed discomfort resulting from movement restrictions following THA, a literature review was conducted. As evidence supporting such restrictions was limited and restrictions could potentially delay recovery, several hospitals decided to eliminate or minimize movement restrictions between 2015 and 2020. Patients in the VBHC program were involved in interpreting the results and in formulating how the updated recommendations were communicated to future patients. Each participating hospital documented its postoperative protocols, and details were collected regarding the dates and methods used to communicate protocol changes to both caregivers and patients.

Patients were assigned to 1 of the 2 groups according to the applicable protocol at the time of surgery. Patients in the strict movement-restriction group received an extensive list of postoperative instructions to avoid specific isolated movements and to use assistive devices. Patients received strict instructions to avoid bending the hip past 90° of flexion, twisting the leg in or out, adducting the hip over the midline, sleeping on their side, crossing their legs, and putting on socks or shoes independently, and were instructed to walk with crutches for 6 weeks, to sleep on their back or to sleep on their side with a pillow between their legs, and to use elevated chairs and elevated toilet seats. Patients in the minimal movement-restriction group either were prescribed no restrictions and instructed to “move as able” or were prescribed minimal movement restrictions. These minimal restrictions included the advice to avoid movements that involved the combination of hip flexion (>90°), internal rotation, and adduction (e.g., crossing their legs while sitting), but patients were otherwise encouraged to “move as able” or “move as your pain allows.” Assistive devices (e.g., crutches, pillows, and elevated chairs) were not mandated but could be used for comfort according to patient preference.

### Data Collection

Patient characteristics, surgical details, and prosthesis characteristics were documented. Hip dislocations within 90 days after THA were recorded as the primary outcome. Additionally, data regarding perioperative and postoperative complications were collected to evaluate comparability between the 2 groups; these included deep postoperative wound infections and fractures within 90 days and unplanned readmissions within 30 days. All data utilized for this study were routinely collected within the VBHC program. Because the data were already collected, all patients who satisfied the eligibility criteria were included in the study^21^.

### Statistical Analysis

The incidence of hip dislocation was compared between the 2 groups with use of a Pearson chi-square test. A multivariable logistic regression analysis was performed to assess the effect of movement restrictions on the incidence of hip dislocations, with correction for potential confounding. Potential confounders were selected on the basis of prior literature and according to clinical reasoning. Early hip dislocations were included in the analysis as the dependent variable, with the respective group (strict movement restrictions[0]/minimal movement restrictions[1]) as the primary independent variable of interest. The analysis also controlled for age at the time of surgery (continuous), sex (male or female), body mass index (BMI; continuous), femoral head size (22 to 28, 32, or 36 mm), and surgical approach (posterolateral, direct lateral, anterior, or anterolateral). In addition, we performed 4 sensitivity analyses to validate our findings: (1) performing a subgroup analysis that repeated the primary analysis in only the patients who underwent the posterolateral approach; (2) exploring the potentially confounding effect of prosthesis fixation, which had not been prespecified as an independent variable in the protocol; (3) exploring the overall effect of time by including the year of surgery in the regression analysis; and (4) performing a propensity score analysis that aimed to correct for potential biases due to differences between the 2 groups. Propensity scores were calculated with use of patient and surgical characteristics (i.e., age, sex, BMI, femoral head size, and surgical approach) and treatment group, and were included in the regression model. Odds ratios (ORs) with 95% confidence intervals (CIs) and p values are reported, and were considered significant if the 95% CI excluded 1. The number needed to treat (NNT) was calculated as 1 divided by the absolute risk difference. Significance was set at 0.05. Analysis was performed with IBM SPSS Statistics 29.

## Results

### Patients

A total of 10,357 patients were included across 7 hospitals. Table 1 presents an overview of each hospital’s movement-restriction policy and the timing of its transition to minimal or no restrictions. One hospital did not change their policy, so all patients managed at this center were included in the group with strict movement restrictions. A total of 7,666 patients were prescribed strict movement restrictions and 2,691 were prescribed minimal movement restrictions following THA. Of these 10,357 patients, 10,352 (99.95%) had data regarding the primary outcome (postoperative hip dislocation) and were included in the analysis. All 5 exclusions due to incomplete hip dislocation data were from the restricted group.

**Table 1:** Hospital Movement-Restriction Protocols, Before and After

Hospital	Protocol Before Change	Date of Protocol Change	Protocol After Change
1	Strict	March 1, 2018	No restrictions*
2	Strict	January 1, 2019	No restrictions*
3	Strict	June 1, 2018	Minimal restrictions†
4	Strict	September 1, 2018	Minimal restrictions†
5	Strict	March 18, 2019	Minimal restrictions†
6	Strict	July 1, 2019	Minimal restrictions for anterior approach‡, strict restrictions for posterolateral and direct lateral approaches
7	Strict	Not applicable	Strict restrictions

* No movement restrictions: move as able/as pain allows.

† Minimal movement restrictions: no combined hip flexion of >90°, internal rotation, adduction; e.g., squatting, crossing the legs while sitting.

‡ Minimal as detailed above in the prior footnote with additional instruction to avoid hyperextending the leg backward while standing and to avoid arching the pelvis up while lying on the back (bridge exercise).

Patient and surgical characteristics are presented in Table 2 and Appendix I. Aside from sex, all variables differed significantly, mainly reflecting a higher use of the posterolateral approach and of 32-mm femoral heads in the minimally restricted group. However, despite these differences, patient characteristics were otherwise comparable, and in both groups the majority of surgeries were performed using the posterolateral approach and a 32-mm femoral head. The groups showed similar rates of deep postoperative wound infection within 90 days (p = 0.770), unplanned readmission within 30 days (p = 0.174), and fracture (0.9% versus 0.5%; p = 0.069).

**Table 2:** Patient and Prosthesis Characteristics and Surgical Approach in the 2 Study Groups*

Variable	Restricted (N = 7,666)	Minimally Restricted (N = 2,691)	P Value
Age *(yr)*	70.4 ± 9.6	69.8 ± 9.9	0.005
BMI *(kg/m*^*2*^*)*	27.8 ± 4.7	27.4 ± 4.9	<0.001
Female sex	5,046 (65.9%)	1,724 (64.1%)	0.09
Femoral head size			<0.001
22-28 mm	2,191 (29.2%)	412 (16.7%)	
32 mm	5,182 (69.1%)	1,961 (79.6%)	
36 mm	130 (1.7%)	90 (3.7%)	
Surgical approach			<0.001
Posterolateral	5,005 (65.4%)	2,164 (80.5%)	
Direct lateral	448 (6.4%)	71 (2.6%)	
Anterior	1,795 (23.5%)	394 (14.7%)	
Anterolateral	361 (4.7%)	59 (2.2%)	
Prosthesis fixation			<0.001
Cemented	4,248 (55.5%)	1,228 (45.7%)	
Uncemented	2,444 (31.9%)	1,364 (50.7%)	
Hybrid	962 (12.6%)	96 (3.6%)	

* Values are given as the mean ± standard deviation or as the count with the percentage in parentheses.

### Hip Dislocations

In the minimally restricted group, 1.93% of patients (52 of 2,691) had an early hip dislocation within 90 days after THA, compared with 1.46% (112 of 7,661) in the restricted group. The incidence of early hip dislocations was not significantly different between the 2 groups (p = 0.093).

Logistic regression analysis, adjusted for potential confounding, showed no significant difference in the odds of early hip dislocation between the two groups (OR, 1.25; 95% CI, 0.86 to 1.82; p = 0.246; Table 3). Although the point-estimate suggests a 25% higher odds of dislocation in the minimally restricted group, this difference was not significant. Significantly lower odds of dislocation were found for female sex (OR, 0.70; 95% CI, 0.50 to 0.99), a 32-mm head size compared with a smaller head size (OR, 0.61; 95% CI, 0.43 to 0.87), and the direct lateral approach compared with the posterolateral approach (OR, 0.12; 95% CI, 0.02 to 0.84). A total of 213 patients would need to be prescribed strict movement restrictions to prevent 1 dislocation (NNT = 213).

**Table 3:** ORs for Early Hip Dislocation According to Movement-Restriction Protocol and Patient, Prosthesis, and Surgical Characteristics*

Variable	OR (95% CI)	P Value
Minimal versus strict movement restriction	1.25 (0.86-1.82)	0.246
Age in yr	1.00 (0.99-1.03)	0.223
BMI in kg/m^2^	1.00 (0.98-1.05)	0.426
Female sex	0.70 (0.50-0.99)	0.044
Femoral head size		
22-28 mm	Reference	
32 mm	0.61 (0.43-0.87)	0.006
36 mm	0.35 (0.08-1.47)	0.152
Surgical approach		
Posterolateral	Reference	
Direct lateral	0.12 (0.02-0.84)	0.033
Anterior	0.99 (0.67-1.48)	0.972
Anterolateral	†	†

* Results were obtained with use of multivariable logistic regression analysis, adjusted for age, sex, BMI, femoral head size, and surgical approach.

† No dislocations occurred in this group; therefore, the OR, CI, and p value could not be calculated.

### Sensitivity Analyses

When assessing only the posterolateral approach, we found a dislocation incidence of 2.03% (44 of 2,164) in the minimally restricted group and 1.50% (75 of 5,000) in the restricted group (p = 0.105). The fully adjusted logistic regression analysis in the posterolateral approach subgroup showed an OR of 1.31 (95% CI, 0.88 to 1.95; p = 0.185) in the minimally restricted group compared with the restricted group. A total of 188 patients would need to be prescribed strict movement restrictions to prevent 1 dislocation (NNT = 188).

The second and third sensitivity analyses showed that the odds of dislocation in the minimally restricted group remained nonsignificant and were similarly close or closer to 1 compared with those in the primary analysis. ORs were 1.26 (95% CI, 0.86 to 1.84; p = 0.233) in the model including prosthesis fixation and 0.96 (95% CI, 0.61 to 1.50; p = 0.850) in the time-adjusted model. Significant associations with the odds of dislocation were noted when comparing cemented versus uncemented fixation and when assessing the year of surgery (Appendix II). Propensity score analysis showed an OR of 1.22 for dislocation in the minimally restricted group as compared to the restricted (95% CI, 0.84 to 1.77; p = 0.308).

## Discussion

The main results of this study confirmed our hypothesis that changing to a protocol with no or minimal movement restrictions following THA would not increase the incidence of early postoperative hip dislocations. We observed a similar incidence of early hip dislocation among patients in the minimally restricted (1.93%) and restricted groups (1.46%; p = 0.093). These findings show that the risk of dislocation might be associated with variables other than postoperative movement protocols.

Our hypothesis was further substantiated after correction for patient and prosthesis characteristics and for surgical factors known to potentially increase the risk of postoperative hip dislocation. The adjusted analysis showed that the use of minimal versus strict movement restrictions had no impact on the odds of hip dislocation (OR, 1.25; 95% CI, 0.86 to 1.82). When assessing only surgeries performed via the posterolateral approach, the difference between the groups was slightly larger but remained nonsignificant (OR, 1.31; 95% CI, 0.88 to 1.95).

The NNT is an estimate of the number of patients required to prevent 1 additional negative outcome through the use of a particular intervention over another. Although our findings were not significant, we calculated the NNT to put the results and potential practical implications into perspective. The analysis showed that a large number of patients would need to be prescribed strict compared with minimal hip precautions to prevent 1 additional dislocation (213 patients for all surgical approaches and 188 patients for the posterolateral approach only). On the basis of the NNT, we question whether the potential prevention of such relatively few dislocations would outweigh the discomfort and delayed recovery of a large number of patients.

Our findings are in line with the existing literature. In 2 separate systematic reviews from 2016, van der Weegen et al. and Smith et al. assessed the results of various surgical approaches in THA and reported no significant increase in hip dislocations with reduced mobility restrictions^22,23^. However, Smith et al. noted that the quality of evidence was very low, with small sample sizes described as one of the most common reasons^23^. A systematic review by Crompton et al. evaluated the results of 6,900 patients from 2 randomized controlled trials and 5 cohort studies. They concluded that a removal or reduction of movement restrictions following THA via the posterior approach did not increase dislocation incidence, with rates of 2.2% in the restricted group and 2.0% in the unrestricted group^24^. A recent large cohort study performed with use of the Danish Hip Arthroplasty Register also showed no clear association between hip precautions and the risk of hip dislocation following posterolateral THA^25^. A secondary analysis of the Danish registry data, solely correcting for femoral head size, showed a potential effect of movement restrictions for THAs performed with 28-mm and 32-mm head sizes; however, after correction for other potential confounders, the results were not significant.

For the variables added as potential confounders in our study, substantially lower odds of dislocation were found for the use of a 32-mm head size compared with a smaller head size, for female sex, and for the direct lateral approach compared with the posterolateral approach. The observed lower dislocation risk for larger head sizes is in line with the existing literature^4^. As a result, guidelines have changed over time, advising the use of larger femoral head sizes. Given the relatively low number of 36-mm femoral heads utilized in our study, no conclusions can be drawn for this subgroup. The literature has shown conflicting results regarding the effects of sex and surgical approach on dislocation risk. Some studies have reported higher odds in female patients, whereas others found no association. In the present study, the odds of postoperative dislocation were higher in male patients. There is also conflicting evidence regarding the association between surgical approach and dislocation risk^26^. Traditionally, it has been believed that a posterior approach increases the risk of dislocation, compared with the direct lateral and anterolateral approaches; however, soft-tissue repair of the capsule and other structures has been shown to play an important role in reducing the risk of dislocation following THA with a posterior approach^4,5,26^.

### Strengths and Limitations

A major strength of this study was the large sample size. Given the low incidence of hip dislocation, large sample sizes are necessary to accurately determine differences in risk between groups. Another strength was the use of real-world data, as the study included all patients who underwent a total hip arthroplasty for OA in 7 Dutch hospitals. Also, we included various surgical approaches to provide a comprehensive assessment of dislocation risk following THA. Traditionally, hospitals have prescribed hip precautions across surgical techniques and not only with use of the posterolateral approach^18^. A uniform set of guidelines is often applied to reduce confusion among caregivers and patients. Because previous studies have indicated a potentially higher dislocation risk with the posterolateral approach, we conducted a sensitivity analysis for this subgroup. Together, these strengths contributed to a study population that was highly representative of the actual patient population.

An important limitation of the present study was that not all patients and caregivers may have adhered to the prescribed protocol for hip precautions. Although the policy change to minimal movement restrictions was communicated to caretakers and patients, it is unknown if all 10,357 patients and their caregivers complied. Patients also may not have complied with strict restrictions because of physical discomfort and/or confusion regarding instructions. Similarly, patients in the minimally restricted group may not have mobilized as instructed. Future prospective studies may benefit from compliance data, such as compliance questionnaires or technology that monitors patient activity. Another limitation of our study was that there were differences in the discontinuation or minimization of movement restrictions between hospitals. Whereas 4 hospitals changed their policy to minimally restrict movement and advised patients, for example, to prevent combined full flexion (>90°), internal rotation, and adduction of the hip, 2 hospitals discontinued all movement restrictions. Additionally, the before-and-after study design is not suited to assess causality. Differences between the groups suggested that, in addition to the change in restriction protocol, surgical preferences such as approach and femoral head size also evolved over time. To adjust for confounding, we included relevant variables in the regression model and performed sensitivity analyses, including for fixation, year of surgery, and propensity-score modeling. Results of these analyses were consistent with the primary analysis and showed no significant association between movement restrictions and dislocation risk. Although we accounted for measurable confounders, sources of residual bias such as incomplete compliance with instructions, secular trends in surgical practice, and unmeasured patient or perioperative factors cannot be fully eliminated. Transparency in reporting these limitations and consistency across models support the robustness of our findings while acknowledging their observational nature. Ideally, a sufficiently powered randomized controlled trial would be conducted, but that would be very time-consuming and costly. We doubt whether such a trial would (and should) still be needed and funded, given the available evidence from smaller randomized controlled trials and large observational data sets, including the present study.

### Conclusions

The results of this before-and-after study show that a protocol change from strict to minimal movement restrictions following THA did not significantly increase the incidence of early hip dislocations. This study, which included >10,000 patients, contributes to the existing evidence suggesting that it is safe to switch to a minimally restrictive postoperative movement protocol.

## Appendix

Supporting material provided by the authors is posted with the online version of this article as a data supplement at jbjs.org (http://links.lww.com/JBJS/J103).
